# Supplementary material for: Deletion of a conserved Gata2 enhancer impairs haemogenic endothelium programming and adult Zebrafish haematopoiesis
Source: Commun Biol. 2020 Feb 13;3:71. doi: 10.1038/s42003-020-0798-3 (PMC7018942; doi:10.1038/s42003-020-0798-3)
Supplement: Supplementary file 2 — Description of Additional Supplementary Files [file 42003_2020_798_MOESM2_ESM.docx]

Description of additional supplementary items

Supplementary Data1 – source data for Figures 1-6 and Supplementary Fig. 1-6

Supplementary Data 2 –list of called ATACseq peaks
