# Supplementary material for: Deletion of a conserved Gata2 enhancer impairs haemogenic endothelium programming and adult Zebrafish haematopoiesis
Source: Commun Biol. 2020 Feb 13;3:71. doi: 10.1038/s42003-020-0798-3 (PMC7018942; doi:10.1038/s42003-020-0798-3)
Supplement: Supplementary file 5 — Reporting Summary [file 42003_2020_798_MOESM5_ESM.pdf]

nature research

Corresponding author(s): Rui Monteiro

Last updated by author(s): Jan 10, 2020

Reporting Summary

Nature Research wishes to improve the reproducibility of the work that we publish. This form provides structure for consistency and transparency in reporting. For further information on Nature Research policies, see Authors & Referees and the Editorial Policy Checklist.

Statistics

For all statistical analyses, confirm that the following items are present in the figure legend, table legend, main text, or Methods section.

n/a	Confirmed	
☐	☒	The exact sample size (*n*) for each experimental group/condition, given as a discrete number and unit of measurement
☐	☒	A statement on whether measurements were taken from distinct samples or whether the same sample was measured repeatedly
☐	☒	The statistical test(s) used AND whether they are one- or two-sided<br>*Only common tests should be described solely by name; describe more complex techniques in the Methods section.*
☒	☐	A description of all covariates tested
☐	☒	A description of any assumptions or corrections, such as tests of normality and adjustment for multiple comparisons
☐	☒	A full description of the statistical parameters including central tendency (e.g. means) or other basic estimates (e.g. regression coefficient) AND variation (e.g. standard deviation) or associated estimates of uncertainty (e.g. confidence intervals)
☐	☒	For null hypothesis testing, the test statistic (e.g. *F*, *t*, *r*) with confidence intervals, effect sizes, degrees of freedom and *P* value noted<br>*Give P values as exact values whenever suitable.*
☒	☐	For Bayesian analysis, information on the choice of priors and Markov chain Monte Carlo settings
☒	☐	For hierarchical and complex designs, identification of the appropriate level for tests and full reporting of outcomes
☒	☐	Estimates of effect sizes (e.g. Cohen's *d*, Pearson's *r*), indicating how they were calculated

Our web collection on statistics for biologists contains articles on many of the points above.

Software and code

Policy information about availability of computer code

Data collection	Sequencing data (ATACseq) was obtained from an Illumina HiSeq 4000 machine in FastQ format and mapped/processed /analysed using the software listed below
Data analysis	BWA aligner 0.7.12<br>Trimmomatic 0.32<br>subread 1.6.2<br>Homer 3.0<br>R diffBinding package

For manuscripts utilizing custom algorithms or software that are central to the research but not yet described in published literature, software must be made available to editors/reviewers. We strongly encourage code deposition in a community repository (e.g. GitHub). See the Nature Research guidelines for submitting code & software for further information.

Data

Policy information about availability of data

All manuscripts must include a data availability statement. This statement should provide the following information, where applicable:

- Accession codes, unique identifiers, or web links for publicly available datasets
- A list of figures that have associated raw data
- A description of any restrictions on data availability

All data generated or analysed during this study are included in this published article (and its supplementary information files). Accession codes for ATACseq are XXXXXXX

Field-specific reporting

Please select the one below that is the best fit for your research. If you are not sure, read the appropriate sections before making your selection.

☒ Life sciences ☐ Behavioural & social sciences ☐ Ecological, evolutionary & environmental sciences

For a reference copy of the document with all sections, see nature.com/documents/nr-reporting-summary-flat.pdf

Life sciences study design

All studies must disclose on these points even when the disclosure is negative.

Sample size	For the ATACseq experiments, we determined that a minimum of 4 samples was sufficient due to the homogeneous genetic background of the zebrafish population and the high numbers of fish pooled for each independent experiment. For confirmatory gene expression experiments using the Biomark platform, sample size was increased to 5-6 independent experiments.
Data exclusions	For ATACseq, four experimental replicates were generated and analysed for consistency by PCA and correlation analysis; two outlying replicates for thr kdrl-GFP+ population were removed, so we analysed 4 kdrl-GFP- and 2 kdrl-GFP+ samples.
Replication	Differential gene expression analysis by in situ hybridization was done on wildtype versus gata2a deltai4/deltai4 mutants. and the genotypes verified after in situ hybridization. Reproducibility of the experimental findings was verified by qPCR in independent experiments in endothelial cells isolated by FACS.
Randomization	For the ATAC experiments, no randomization was possible. For the ISH experiments, embryos of mixed genotypes were stained together and then randomly imaged and genotyped.
Blinding	Quantification of the ISH signals in embryos were done blindly (prior to genotyping). Analysis of the adult WKM was not blinded as animals were genotyped before being allowed to grow to adulthood.

Reporting for specific materials, systems and methods

We require information from authors about some types of materials, experimental systems and methods used in many studies. Here, indicate whether each material, system or method listed is relevant to your study. If you are not sure if a list item applies to your research, read the appropriate section before selecting a response.

Materials & experimental systems

n/a	Involved in the study
☐	☒ Antibodies
☒	☐ Eukaryotic cell lines
☒	☐ Palaeontology
☐	☒ Animals and other organisms
☒	☐ Human research participants
☒	☐ Clinical data

Methods

n/a	Involved in the study
☒	☐ ChIP-seq
☐	☒ Flow cytometry
☒	☐ MRI-based neuroimaging

Antibodies

Antibodies used	Antibodies for in situ hybridization: Anti-digoxigenin-AP, Fab fragments (11093274910, SIGMA) and Anti-Fluorescein-POD, Fab fragments (11426346910, SIGMA)<br>Antibodies for immunochemistry: rabbit anti-GFP (A-11122, Invitrogen) and goat anti-rabbit conjugated Alexa-488 (A32731, Invitrogen)
Validation	All validation of the antibodies used can be found in the manufacturer's website

Animals and other organisms

Policy information about studies involving animals; ARRIVE guidelines recommended for reporting animal research

Laboratory animals	Zebrafish (danio rerio), males and females, AB strain. Tg(-6.0itga2b:EGFP)la2, Tg(kdrl:GFP)s843, Tg(gata2a -i4:GFP) and gata2a deltai4/deltai4 mutants. Fish were analyzed during embryogenesis (around 24-96 hours post fertilization) and as adults (>3 months post fertilization).
Wild animals	Study did not involve wild animals
Field-collected samples	Study did not involve samples collected from the field

Ethics oversight	All experiments were performed under a Project license approved by the Home Office, UK, under the Animals (Scientific Procedures) Act 1986.

Note that full information on the approval of the study protocol must also be provided in the manuscript.

Flow Cytometry

Plots

Confirm that:

☒ The axis labels state the marker and fluorochrome used (e.g. CD4-FITC).

☒ The axis scales are clearly visible. Include numbers along axes only for bottom left plot of group (a 'group' is an analysis of identical markers).

☒ All plots are contour plots with outliers or pseudocolor plots.

☒ A numerical value for number of cells or percentage (with statistics) is provided.

Methodology

Sample preparation	ATACseq: Tg(kdrl:GFP)s843 embryos were dissociated for FACS at 26-27hpf to collect kdrl + and kdrl - cell populations (40,000-50,000 cells each). They were processed for ATAC library preparation using optimised standard protocol 26. Briefly, after sorting into Hanks' solution (1xHBSS, 0.25% BSA, 10mM HEPES pH8), the cells were spun down at 500g at 4°C, washed with ice-cold PBS and resuspended in 50µl cold Lysis Buffer (10mM Tris-HCl, 10mM NaCl, 3mM MgCl2, 0.1% IGEPAL, pH 7.4). The nuclei were pelleted for 10min. at 500g at 4°C and ressuspended in the TD Buffer with Tn5 Transposase (Illumina), scaling the amounts of reagents accordingly to the number of sorted cells. The transposition reaction lasted 30min. at 37°C. The DNA was purified with PCR Purification MinElute Kit (QIAGEN). In parallel, transposase-untreated genomic DNA from kdrl+ cells was purified with the DNeasy® Blood & Tissue Kit (QIAGEN). The samples were amplified with appropriate Customized Nextera primers 26 in NEBNext High-Fidelity 2x PCR Master Mix (NEB). The libraries were purified with PCR Purification MinElute Kit (QIAGEN) and Agencourt AMPure XP beads (Beckmann Coulter). The quality of each library was verified using D1000 ScreenTape System (Agilent). Four biological replicas of the libraries were quantified with the KAPA Library Quantification Kit for Illumina® platforms (KAPA Biosystems). The libraries were pooled (including the Tn5 untreated control), diluted to 1ng/µl and sequenced using 75bp paired-end reads on Illumina HiSeq 4000 (Wellcome Trust Centre for Human Genetics, Oxford).
Instrument	BD FACS Aria Fusion system
Software	FACSDiva v8.0.1 Software
Cell population abundance	kdrl+ (high): ~1-2%; kdrl-: ~94-95%
Gating strategy	We first sorted for live/dead cells using a Hoechst(DEAD) dye, then excluded doublets. From these we set the gates for negative and positive GFP expression with wildtype and single transgenics as single-colour controls. To identify the gating strategy for isolation of endothelial cells, we split the GFP+ gate into two sub-gates (low and high). We determined that the best enrichment of endothelial markers was found in the kdrl GFPhigh gate and used that for further experiments (ATACse and Biomark qPCR).

☒ Tick this box to confirm that a figure exemplifying the gating strategy is provided in the Supplementary Information.
